# Flow-controlled One-lung Ventilation During Carinal Sleeve Reconstruction: Anaesthetic Management and Technical Feasibility

**DOI:** 10.4274/TJAR.2026.262398

**Published:** 2026-04-15

**Authors:** Cansu Kılınç Berktaş, Emine Şeyda Teloğlu, Funda Gümüş Özcan, Ece Yasemin Demirkol

**Affiliations:** 1 University of Health Sciences Türkiye, Başakşehir Çam and Sakura City Hospital, Clinic of Anaesthesiology and Reanimation, İstanbul, Türkiye; 2 University of Health Sciences Türkiye, Başakşehir Çam and Sakura City Hospital, Clinic of Thoracic Surgery, İstanbul, Türkiye

**Keywords:** Airway management/methods, anaesthesia, thoracic, flow-controlled ventilation, positive-pressure respiration, tracheal neoplasms/surgery, ventilation, one-lung

## Abstract

Carinal sleeve resection with reconstruction represents one of the most challenging thoracic surgical procedures and requires meticulous anaesthetic management to maintain adequate gas exchange within a shared and surgically interrupted airway while preserving an unobstructed surgical field. Conventional ventilation strategies, including cross-field ventilation and high-frequency jet ventilation, may interfere with surgical exposure or result in unstable ventilation. We describe the anaesthetic management of a 64-year-old male patient in whom flow-controlled ventilation- one-lung ventilation (FCV-OLV) was employed during carinal sleeve lobectomy with reconstruction. Following initial double-lumen tube ventilation, FCV was established using a Tritube^®^ connected to an Evone^®^ ventilator and maintained throughout airway reconstruction. Ventilation was achieved without interruptions, apneic periods, or cross-field ventilation. Oxygenation and carbon dioxide elimination remained stable during 180 minutes of FCV, while the small-lumen tube provided optimal surgical exposure. The patient was extubated uneventfully at the end of surgery and experienced an uncomplicated postoperative course. This case highlights the feasibility of flow-controlled OLV as an anaesthetic strategy during complex airway reconstruction. FCV-OLV enabled uninterrupted ventilation, stable gas exchange, and excellent surgical conditions without the need for extracorporeal support or alternative ventilation techniques

**Main Points**

• Flow-controlled ventilation (FCV) can provide uninterrupted gas exchange during complex airway reconstruction.

• FCV- one-lung ventilation may improve surgical exposure by allowing the use of a small-lumen ventilation tube.

• This technique can be applied as a rescue strategy when unexpected carinal involvement occurs.

• FCV may reduce the need for cross-field ventilation or extracorporeal support in selected cases.

## Introduction

Involvement of the carina by lung or tracheal tumors is uncommon and remains one of the most challenging scenarios in thoracic surgery. Despite advances in bronchial sleeve lobectomy and tracheal surgery, tumors invading the carina are rarely amenable to resection and require highly specialized surgical and anaesthetic management.^1, 2^ From an anaesthetic perspective, the principal challenge lies in maintaining adequate oxygenation and carbon dioxide elimination while preserving an unobstructed surgical field within a shared and surgically interrupted airway.

Several ventilation strategies have been described during carinal reconstruction, including cross-field ventilation, high-frequency jet ventilation (HFJV), and extracorporeal membrane oxygenation (ECMO).^2, 3^ However, each technique has important limitations. Cross-field ventilation may interfere with surgical exposure and workflow, HFJV has been associated with inadequate carbon dioxide clearance, air trapping, and aerosol generation, and ECMO introduces additional complexity, cost, and anticoagulation-related risks.^1, 3^

Flow-controlled ventilation (FCV) is a relatively novel ventilation technique that delivers a constant and controlled gas flow during both inspiration and active expiration. When applied through small-lumen tubes such as the Tritube^®^, FCV enables continuous ventilation while minimizing airway obstruction. Experimental and clinical studies have demonstrated that FCV may improve oxygenation, reduce peak airway pressures, and decrease mechanical power compared with conventional ventilation modes.^4, 5, 6, 7^ More recent investigations have suggested that FCV may be particularly advantageous during one-lung ventilation and airway surgery by providing stable gas exchange and improved surgical conditions.^5, 6^

In this report, we describe the anaesthetic management of a patient undergoing carinal sleeve lobectomy with reconstruction using FCV-OLV. The purpose of this case is to highlight the feasibility of FCV-OLV as an alternative airway management strategy in complex airway reconstruction, focusing on anaesthetic considerations rather than surgical or oncologic outcomes.

## Case Report

A 64-year-old male patient (American Society of Anesthesiologist Physical Status II) with a predicted body weight (PBW) of 81.5 kg (calculated according to standard formulas for males) was scheduled for right upper lobectomy via thoracotomy due to suspected lung malignancy. Preoperative evaluation revealed adequate cardiopulmonary reserve, with pulmonary function testing showing an forced expiratory volume in 1 second/forced vital capacity ratio of 85%. Standard monitoring was applied, including electrocardiography, pulse oximetry, invasive arterial blood pressure monitoring via a left radial arterial line, and end-tidal carbon dioxide monitoring. An 8F central venous catheter was placed in the right internal jugular vein. Written informed consent was obtained from the patient for publication of this case report and accompanying images.

General anaesthesia was induced with propofol (2 mg kg^-1^), fentanyl (2 µg kg-^1^), and rocuronium (0.6 mg kg^-1^). The trachea was intubated with a 37F left-sided double-lumen tube (DLT), and mechanical ventilation was initiated in pressure-controlled mode. Initial intraoperative conditions were stable.

During surgical exploration, frozen-section analysis revealed tumor involvement of the carina, so the initially planned right upper lobectomy was converted to a carinal sleeve lobectomy with reconstruction. Following this unexpected intraoperative finding, conventional ventilation strategies were no longer feasible without compromising the surgical field. Therefore, FCV was introduced intraoperatively as a rescue strategy to maintain adequate ventilation while optimizing surgical exposure.

At this stage, the DLT was withdrawn to the tracheal level, and temporary ventilation was achieved via a 7.0 mm standard endotracheal tube surgically inserted into the left main bronchus. Subsequently, a Tritube^®^ was introduced retrogradely through the surgical field, retrieved orally, and connected to an Evone^®^ ventilator. The cuff of the Tritube^®^ was positioned in the left main bronchus and remained in place throughout airway reconstruction, allowing continuous ventilation without interruption or apneic periods.

From the initiation of FCV until the end of the procedure, anaesthesia was maintained with total intravenous anaesthesia (TIVA), administered as continuous infusions of propofol and remifentanil. The depth of anaesthesia was titrated to maintain a SedLine^®^ patient state index between 25 and 50. Propofol infusion rates ranged between 6-10 mg kg^-1^ h^-1^ and remifentanil infusion rates between 0.05-0.1 µg kg^-1^ min^-1^, adjusted according to hemodynamic and electroencephalographic parameters. TIVA was preferred because FCV was delivered via a separate ventilator system without connection to a conventional anaesthesia machine vaporizer. No ventilatory complications occurred during FCV, and hemodynamic parameters remained stable without the need for vasoactive support.

Ventilation was maintained using FCV with an inspired oxygen fraction of 1.0, a constant flow of 12 L min^-1^, an inspiratory-to-expiratory ratio of 1:1, and an end-expiratory pressure of 8 mbar. Peak inspiratory pressure was approximately 19 mbar, with an average tidal volume of 310 mL (corresponding to 3.8 mL kg^-^1 PBW) and a respiratory rate of 20 breaths per minute. End-tidal carbon dioxide values remained at approximately 31 mmHg. Arterial blood gas analyses were performed intermittently during FCV, and the results remained within acceptable limits, with no episodes of significant hypercapnia or respiratory acidosis.

FCV was maintained for 180 minutes during tracheal, left main bronchial, and intermediate bronchial anastomoses. The tube position was continuously monitored throughout airway reconstruction, and no displacement or loss of the airway occurred. Hemodynamic parameters remained stable, and no episodes of desaturation were observed.

The total surgical duration was 270 minutes. After completion of reconstruction and confirmation of airway integrity, the Tritube^® ^was withdrawn, and the patient was extubated uneventfully at the end of surgery. He was transferred to the intensive care unit for postoperative observation, remained stable during a 16-hour stay, and was subsequently transferred to the ward. The patient was discharged on postoperative day 8 without complications. Follow-up fiberoptic bronchoscopy performed one month later demonstrated a well-healed anastomosis without evidence of stenosis or leakage.

## Discussion

Carinal sleeve resection with reconstruction is one of the most demanding scenarios in thoracic anaesthesia because the airway is shared and surgically interrupted, and continuous ventilation is required without compromising surgical exposure. Despite advances in surgical techniques, tumors involving the carina remain rare and technically challenging, requiring close coordination between the surgical and anaesthetic teams.^1, 2^ From an anaesthetic perspective, maintaining adequate oxygenation and carbon dioxide elimination while preserving an unobstructed operative field is the principal challenge.

Several ventilation strategies have been described during carinal surgery, including cross-field ventilation, HFJV, and ECMO.^2, 3^ However, each approach has inherent limitations. Cross-field ventilation may interfere with surgical workflow and visualization, HFJV has been associated with inadequate carbon dioxide elimination, air trapping, and aerosol generation, and ECMO introduces additional complexity, cost, and risks related to anticoagulation and cannulation.^1, 3^ These limitations emphasize the need for alternative ventilation strategies that allow uninterrupted ventilation while maintaining optimal surgical conditions.

FCV was not planned preoperatively but was introduced intraoperatively as a rescue strategy after frozen-section analysis confirmed carinal involvement, and the procedure was converted to carinal sleeve reconstruction. This scenario highlights an important clinical advantage of FCV: its feasibility in unexpected intraoperative situations requiring rapid adaptation of airway management. FCV enabled uninterrupted one-lung ventilation throughout airway reconstruction without the need for cross-field ventilation, apneic periods, or extracorporeal support, thereby maintaining stable gas exchange and optimal surgical exposure. In addition, when FCV is delivered via a standalone ventilator system, TIVA may be preferable, as it avoids fluctuations in anaesthetic concentration and allows a stable anaesthetic depth independent of the anaesthesia workstation.

From a physiological standpoint, FCV differs fundamentally from conventional ventilation modes. It delivers a constant and controlled gas flow during both inspiration and active expiration, allowing more precise regulation of airway pressures and gas exchange.^4, 7^ In FCV, tidal volume is not set as a predefined target but results from the interaction between applied flow, pressure limits, and patient-specific respiratory mechanics.^8, 9, 10^ Experimental and clinical studies have demonstrated that FCV may improve oxygenation, reduce peak airway pressures, and decrease mechanical power compared with pressure- or volume-controlled ventilation, particularly during one-lung ventilation.^5, 6, 8, 10, 11, 12^ In our case, the selected end-expiratory pressure facilitated maintenance of alveolar recruitment during prolonged one-lung ventilation without excessive airway pressures, resulting in stable oxygenation and normocapnia.

**Figure 1:**
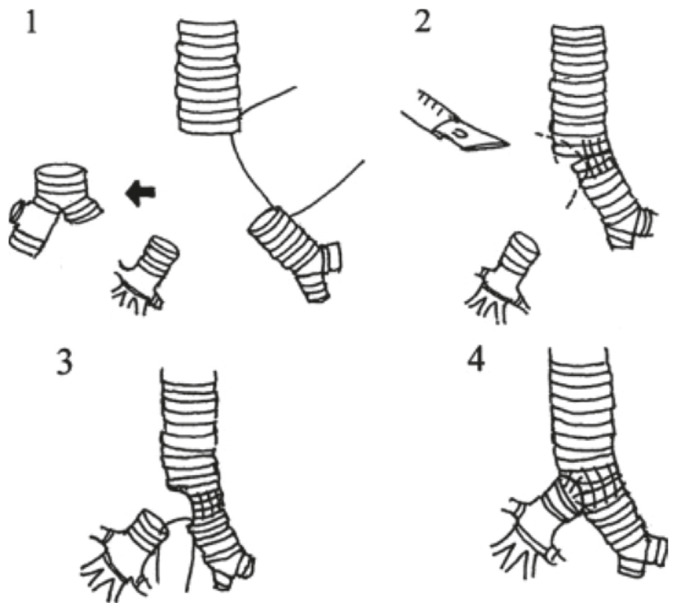
Carinal reconstruction technique used in this case. Adapted from Yamamoto et al. ^15^ A novel carinal reconstruction technique. (1) After the tracheal carina is resected, two thirds of the circumference of the trachea and the left main bronchus are anastomosed. (2) The remaining one third of the circumference is trimmed to create an oval-shape orifice to which the right bronchus is anastomosed. (3) The right bronchus is anastomosed to this trimmed orifice in end-to-side fashion.

Beyond gas exchange, surgical exposure is a critical determinant of success during carinal reconstruction. Using a small-lumen tube, such as the Tritube®, in combination with FCV provided excellent visualization of the operative field and eliminated the need for repeated tube manipulation. The Tritube^®^ is a small-lumen endotracheal tube designed for use with dedicated FCV systems, and its small outer diameter facilitates surgical access during airway reconstruction. Although small-lumen tubes carry a potential risk of obstruction by blood or secretions, careful monitoring and the ability to flush and suction through the tube may facilitate management if needed. Previous clinical studies have reported similar advantages of FCV in airway and upper thoracic surgery, including improved surgical comfort, reduced aerosolization, and uninterrupted ventilation.^4, 7, 11, 13^ Our experience is consistent with these findings and extends them by demonstrating the feasibility of FCV as a rescue strategy during complex carinal reconstruction. From a surgical perspective, various techniques for carinal reconstruction have been described, each associated with specific technical challenges and potential complications.^14, 15^ The surgical reconstruction technique used in this case is illustrated in Figure 1.^15^ Although surgical outcomes depend primarily on meticulous technique and preservation of vascular supply, anaesthetic strategies that ensure stable ventilation and optimal exposure play a crucial supportive role in achieving successful reconstruction.^1, 14^ The present case underscores the importance of adaptable anaesthetic management in facilitating complex airway surgery.

Ventilatory and hemodynamic parameters during FCV are summarized in Table 1, together with commonly reported clinical ranges for FCV, to facilitate interpretation.

### Study Limitations

This report describes a single case and therefore cannot be generalized. The absence of comparative data precludes conclusions regarding superiority over other ventilation strategies. Additionally, outcomes may be influenced by institutional experience with FCV and close interdisciplinary collaboration. Accordingly, the observations presented here should be considered hypothesis-generating.

### Clinical Implications

This case suggests that flow-controlled one-lung ventilation may be considered when unexpected intraoperative findings necessitate uninterrupted ventilation and optimal surgical exposure during complex airway reconstruction. FCV may serve as a valuable addition to the anaesthesiologis’s armamentarium in selected high-risk thoracic procedures.

## Conclusion

Flow-controlled one-lung ventilation enabled stable oxygenation, effective carbon dioxide elimination, and uninterrupted ventilation during carinal sleeve resection with reconstruction in the present case. Introduced intraoperatively as a rescue strategy following unexpected carinal involvement, FCV provided optimal surgical exposure without the need for cross-field ventilation or extracorporeal support. This case highlights the feasibility of FCV as an adaptable airway management option in complex thoracic surgery when conventional ventilation techniques are insufficient. Further clinical experience and comparative studies are needed to better define its role in airway reconstruction.

## Ethics

**Informed Consent:** Written informed consent was obtained from the patient for publication of this case report and accompanying images.

## Figures and Tables

**Table 1. Ventilatory and Hemodynamic Parameters During 180 Minutes of Flow-controlled One-lung Ventilation:** 

**Parameter**	**Observed value**	Typical clinical range in FCV*
FiO_2_	1.0	As clinically indicated
Constant flow	12 L min^-1^	~8-16 L min^-1^
I:E ratio	1:1	1:1 commonly applied
End-expiratory pressure	8 mbar	5-10 mbar (adjusted individually)
Peak inspiratory pressure	~19 mbar	Adjusted to lung mechanics
Respiratory rate	20 breaths/min	Titrated for CO_2_ control
Tidal volume	310 mL (3.8 mL kg^-1^ PBW)	Not preset; depends on flow and pressure settings
End-tidal CO_2_	~31 mmHg	30-45 mmHg targeted range
Duration of FCV	180 min	-
Hemodynamic stability	Stable; no vasoactive support required	-

*: Typical ranges reflect values commonly reported in clinical studies of flow-controlled ventilation and are not fixed manufacturer-recommended settings

FiO_2_, fraction of inspired oxygen; CO_2_, carbon dioxid*; *PBW, predicted body weight; FCV, flow-controlled ventilation
